# Destructive Effects of Slag from Municipal Waste Incineration Plants on Cement Composites

**DOI:** 10.3390/ma17225559

**Published:** 2024-11-14

**Authors:** Marta Sybis, Jacek Mądrawski, Wojciech Kostrzewski, Emilia Konował, Zbigniew Walczak, Ireneusz Laks

**Affiliations:** 1 Department of Construction and Geoengineering, Poznan University of Life Sciences, Piątkowska 94 E, 60-649 Poznan, Poland; jacek.madrawski@up.poznan.pl (J.M.); wojciech.kostrzewski@up.poznan.pl (W.K.); zbigniew.walczak@up.poznan.pl (Z.W.); ireneusz.laks@up.poznan.pl (I.L.); 2 Institute of Chemistry and Technical Electrochemistry, Poznan University of Technology, Berdychowo 4, 60-965 Poznan, Poland; emilia.konowal@put.poznan.pl

**Keywords:** slag, aluminum, expansion concrete, hydrogen

## Abstract

The increasing production of solid waste and the scarcity of natural aggregates as a matter of fact have made waste recycling a necessity. One such waste, which is generated in large quantities, is slag. However, slag from incineration plants may contain harmful elements that adversely affect the physical, chemical and mechanical properties of cement composites. This study presents laboratory research results on the effect of slag from the Poznan Municipal Waste Thermal Conversion Plant (Poland) on the physicochemical properties of cement composites. The samples were analyzed by scanning electron microscopy (SEM), energy dispersive X-ray spectroscopy (EDS), X-ray diffraction (XRD) and X-ray photoelectron spectroscopy (XPS). It was shown that the slag analyzed contained significant amounts of aluminum, which had a direct effect on the structure of the concrete. An example of this influence is the release of hydrogen during reactions, which causes swelling and cracking of the concrete and reduces its mechanical strength. The authors emphasize that waste aggregate (slag) can be effectively used in the production of concrete after appropriate processing that reduces the risk of adverse effects.

## 1. Introduction

The continuous development of the economy and the constant increase in consumption have led to a significant increase in the production of solid waste, both municipal and construction waste, in recent years [1,2]. In 2020, the total amount of waste generated in EU countries was more than 2.15 billion Mg [3]. The accumulation of waste and the lack of available storage facilities, coupled with concerns about the natural environment, have prompted the search for innovative solutions to significantly reduce the amount of waste generated. However, slag from incineration plants may contain toxic elements such as lead, cadmium, arsenic, or mercury [4,5]. The presence of these substances poses potential risks to human health and the environment, especially over the long term when materials containing such additives are used in construction [6]. Therefore, it is crucial to understand and assess the risks associated with using slag in concrete. The literature highlights the importance of appropriate processing and stabilization of slag to minimize the content of harmful substances and prevent their potential negative impact [2,7,8]. Thermal waste conversion can be carried out in municipal waste incineration plants. However, in this case too, combustion waste is generated in the form of slag and bottom ashes [9,10]. Approximately 0.25 Mg of slag and about 0.075 Mg of fly ash, dust from dust collection, filter cakes, and gypsum from flue gas cleaning processes are produced per ton of municipal solid waste (MSW) incinerated [11]. Another problem is the scarcity of natural aggregates, which are necessary for use in construction, mainly for concrete production.

Concrete is a composite material, essentially consisting of various components, including binding materials, water, aggregates, and admixtures. Among these components, aggregate plays a particularly significant role in concrete, occupying the largest volume, which is approximately 60–75% of the total volume of concrete. It is irreplaceable in all construction work [12,13]. The versatility of concrete as a construction material is attributable to its high strength, low production and maintenance costs, resistance to weather conditions, and excellent structural performance. Economically, concrete is a highly advantageous material compared to other construction materials. Furthermore, the concurrent growth of the economy and population has led to a significant increase in the rate of industrialization and urbanization, making concrete a highly desirable material. However, its production also results in the intensive consumption of natural resources and can potentially lead to unsustainable development based on, among other things, utility maximization [14]. Concrete plays a crucial role in the country’s economic development due to its high volumetric use. Approximately 20 billion tons of raw materials (coarse aggregate) are utilized annually [15].

The solution to the extensive use of natural resources is recycling. Unfortunately, waste aggregates are composed of many characteristics that determine their direct use. Most slag contains admixtures of toxic elements such as As, Pb, Cd, Co, Al, Cr, or Ni. Since these substances can be leached from slag to some extent, potential environmental hazards cannot always be ruled out. Incinerator residues contain hazardous substances such as heavy metals and dioxins. In order to recycle these hazardous materials, long-term safety aspects must also be considered for the sake of future generations. This issue has been addressed by scientific institutions around the world for many years, and research results have been presented in numerous publications [1,16,17,18,19].

The authors of article [20] conducted research on the development of concrete formulas using bottom ash and APC (air pollution control fly ash). The authors of article [21] also studied cement bonding with incinerator waste. The research described in article [22] focused on the use of alkali-activated IFA (incinerator fly ash) as structural materials for civil engineering applications. During the preparation of concretes based on incinerator aggregate, reactions between the cement and the aluminum or zinc contained in the waste are particularly undesirable. Aluminum reacting with water forms tetra- or hexahydroxy aluminates accompanied by the release of hydrogen. The causes and course of these chemical reactions have been described in detail in the literature [10,23]. It is known that these reactions cause the phenomenon of swelling and even cracking of concrete.

On the other hand, a publication [24] indicated that the structure of MSWI (municipal solid waste incineration) slag is loose and irregular, with SiO_2_ as the main component. The presence of SiO_2_ and Al_2_O_3_ in fly ash and slag allows for the participation of these components in the hydration reaction of cement, thereby increasing the strength of concrete. Thus, it is confirmed that fly ash and slag from waste incineration can replace cement and aggregate in suitable proportions. This method effectively addresses the shortage of solid waste landfill space. To mitigate the swelling and cracking of concrete and the release of hydrogen gas, slag and ash from incineration plants are typically seasoned and processed before being used for construction materials.

Depending on the initial composition and future use, waste processing can take various forms, including seasoning, washing, sodium hydroxide treatment, heavy metal removal, and vitrification [6]. A number of studies highlight the importance of such treatments, or at least pre-washing of the acquired slag. A significant number of studies indicate that only the processed (cleaned) combustion product has the appropriate chemical and physical properties to ensure the expected quality of concrete [25,26]. However, the materials obtained by the authors, although environmentally friendly, were often characterized by low strength, usually due to a large amount of impurities contained in bottom ashes. Consequently, it is necessary to reduce the quantity of aluminum and glass present in incineration waste, as well as to minimize the release of potentially harmful substances into the environment [27,28,29].

The objective of this study was to examine the impact of slag from municipal waste incineration plants as a recycled aggregate on the properties and behavior of cement composites. The authors identified the negative effect of aluminum present in the slag on the structure of concrete and discussed the issue of hydrogen emitted in significant quantities.

## 2. Materials

The waste material under analysis was slag, a byproduct of the municipal waste incinerator (SOK). According to estimates, the incinerator processes 210,000 tons of waste from Poznan and nine neighboring municipalities on an annual basis. Each day, 27 tons of fine and coarse slag remain on the grates, which amounts to approximately 52,416–57,166 Mg of raw slag per year and 6916–7380 Mg of solid waste from waste gas treatment (data for 2020–2022) [30].

**Figure 1:**
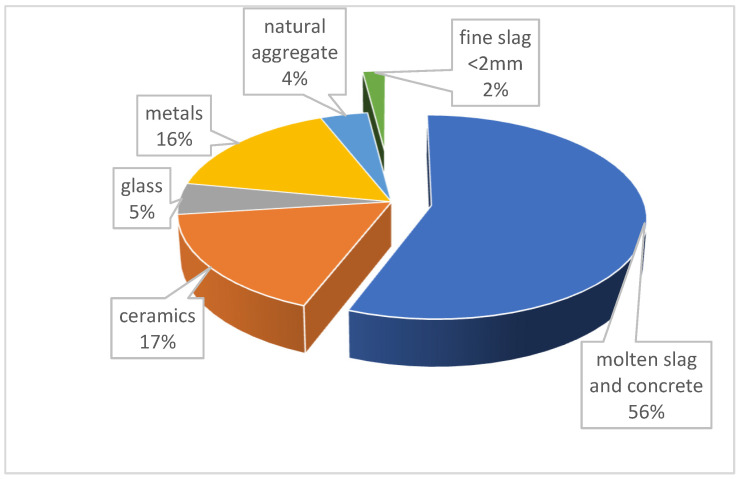
Material composition of coarse slag.

**Figure 2:**
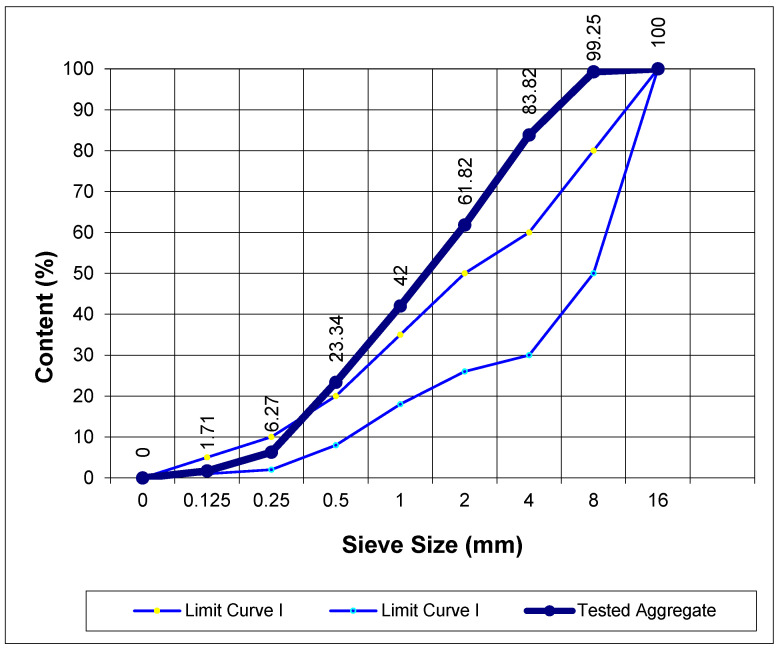
Slag grain size curve.

In order to determine the technical characteristics of the aggregate, the dry sieving method [31] (PN-EN 206+A2:2021-08) was applied, using a set of standard sieves. The composition of the coarse fraction slag is shown in Figure 1, and the grain size curve is shown in Figure 2.

The composition of slag can vary significantly depending on the origin and type of waste disposed of or incinerated. An analysis of slag composition showed significant differences compared to data in the literature, such as those presented in [29]. The glass content is at a similar level as reported by the literature sources, but the content of construction waste, especially ceramics and concrete rubble, is significantly higher. The slag is characterized by a water absorption of 17.9% and a loose bulk density of 1230 kg/m^3^.

Optical Emission Spectroscopy (OES), Energy Dispersive Spectroscopy (EDS), and X-ray diffraction (XRD) were employed to gain a comprehensive understanding of the composition and behavior of the slag used in cementitious composites.

**Figure 3:**
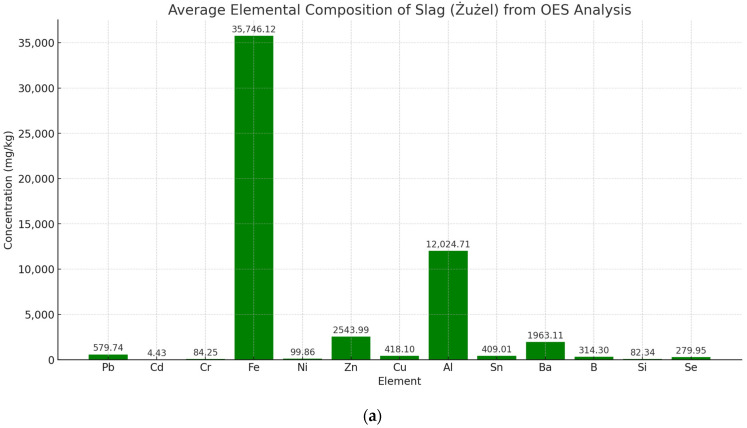

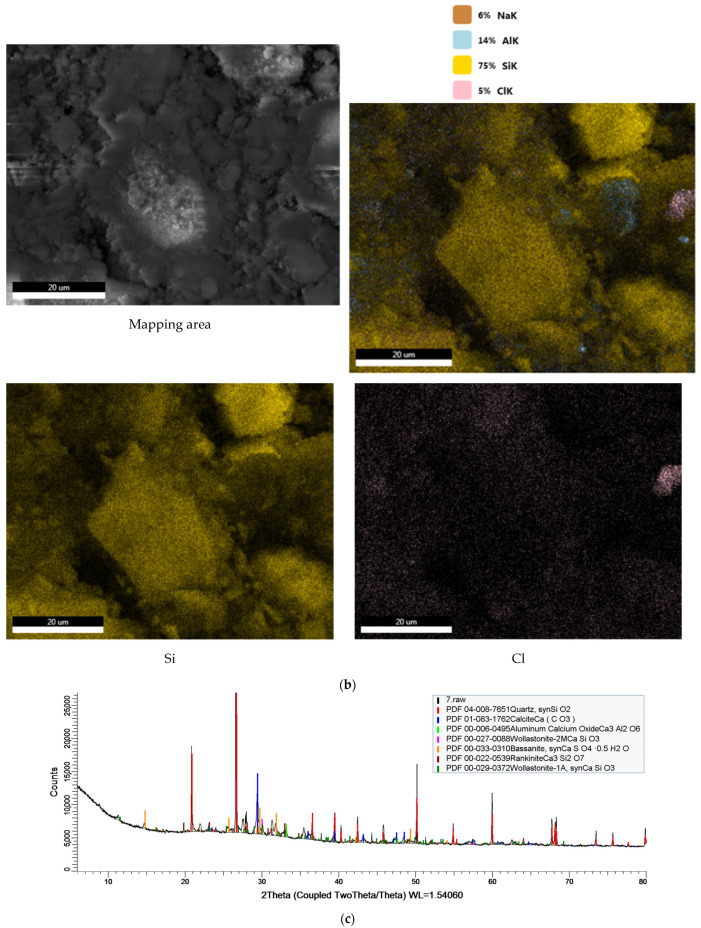
(**a**) Average element composition of slag from OES analysis. (**b**) Elemental composition of the slag as determined by SEM/EDS microscopy (EDS-mapping). (**c**) Diffractogram for slag.

The OES analysis of the slag samples, as shown in Figure 3a, reveals significant concentrations of key elements such as iron (Fe) (35,746.12 mg/kg), aluminum (Al) (12,024.71 mg/kg), and zinc (Zn) (2543.99 mg/kg), with lead (Pb) and chromium (Cr) also present at notable levels. Iron dominates the composition, which is consistent with the slag’s origin from the municipal waste incineration process, where metals accumulate during combustion. The elevated aluminum content raises concerns regarding its reactivity when used in cement composites. In particular, aluminum reacts with water to form hydroxides and release hydrogen gas, which can lead to swelling and cracking of the concrete, posing a serious risk to the material’s structural integrity. Additionally, the presence of heavy metals such as lead (Pb) (579.74 mg/kg) and zinc (Zn) poses potential environmental risks, especially in terms of leaching into soil and groundwater if the slag is not sufficiently treated prior to use.

The EDS (Energy Dispersive Spectroscopy) analysis provides a more localized view of the elemental composition of the slag, offering insights into the distribution of specific elements. The analyses were performed in accordance with EN ISO 22309:2011 [32]. The EDS mapping, shown in Figure 3b highlights the presence of silicon (Si), aluminum (Al), sodium (Na), and chlorine (Cl) across the slag sample. Silicon, consistent with the silicate nature of the slag, is the most abundant element. The significant presence of aluminum confirms the findings from the OES analysis and further emphasizes the potential chemical reactivity of the slag. However, it is important to note that EDS provides only a qualitative analysis of a small area and is not fully representative of the entire sample.

In order to gain a more comprehensive insight into the structure of the slag, an X-ray diffraction (XRD) analysis was conducted [33]. As illustrated in Figure 3c, the XRD diffractogram enables the identification of the crystalline phases present in the slag. The principal phases identified are mono-, di-, and tri-calcium silicates, aluminum oxides, and calcite. These phases are typical of slag from municipal waste incineration plants, where calcium silicates play a critical role in enhancing the mechanical properties of cement composites. The presence of calcite also suggests the potential for carbonation, which could influence the long-term mechanical properties of the concrete. The identification of calcium silicates through XRD supports the use of slag as a partial replacement for natural aggregates in concrete, provided that the material is processed to reduce the risks associated with aluminum reactivity.

The integrated results from the ESA, EDS, and XRD analyses offer a comprehensive representation of the slag’s composition and its potential impact on cement composites. The high concentrations of iron, aluminum and zinc, in conjunction with the crystalline phases identified by XRD, indicate that the slag has the potential to enhance the strength and durability of cement composites. However, the risks associated with aluminum reactivity and heavy metal leaching must be mitigated through the implementation of appropriate processing techniques, such as washing or seasoning, prior to the safe and effective utilization of the slag in construction materials.

### Portland Cement

**Figure 4:**
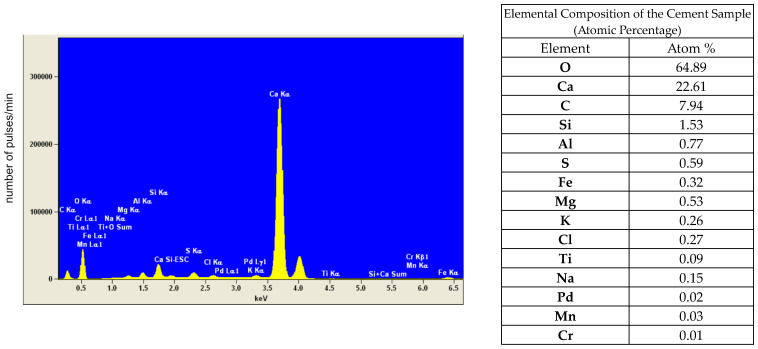
Elemental EDS analysis of cement.

All tests were conducted using Portland cement of the CEM I 42.5N class, in accordance with the European standard PN-EN 196-1:2016-07 [34]. The cement was obtained from the Heidelberg Materials factory. Elemental microanalysis of the cement was performed using an SEM microscope and an EDS detector. The results are presented in Figure 4.

A chemical analysis of the cement sample revealed the presence of calcium, oxygen, carbon, silicon, aluminum, and sulfur, which is consistent with the composition of clinker and gypsum, the primary components of Portland cement. The presence of elements below 0.5% can be considered insignificant, or it may be attributed to the measurement error of the instrument.

## 3. Methodology

The scope of the research carried out included studies on the physical and chemical characteristics of cement mortars, as well as studies to understand the structure and elemental composition of the produced cement composite (XRD, FT-IR, SEM + EDS). The research also included the issue of gas emitted from the concrete, as well as learning the cause of its expansion. The research was divided into 2 stages:

- Gas emission study: Two cubes, 150 mm × 150 mm × 150 mm, to determine the amount and intensity of gas emission by concrete with slag additives and the variation in intensity over time,
- Chemical composition tests: for each of the tests (XRD, FT-IR, SEM + EDS), beams of 40 mm × 40 mm × 160 mm (one according to recipe B of Table 1 and one according to recipe C) aimed at determining the chemical composition and causes of swelling of the samples.

### 3.1. The Formula of the Tested Cement Mortars

For the study of chemical composition analysis and swelling of the samples, the recipe of cement mortars was prepared (A, B of Table 1). Analyses of gas release measurements were performed on 150 mm × 150 mm × 150 mm blocks prepared based on the recipe in C of Table 1.

The SOK milled slag in recipe B was ground in a laboratory ball mill, equipped with steel balls with a total mass of 20 kg. The sieve analysis of the milled slag resulted in the following distribution:

- 0.5–1 mm: 0.4%
- 0.25–0.5 mm: 1.7%
- 0.125–0.25 mm: 55.3%
- <0.125 mm: 42.6%

This milling process ensured a well-defined particle size distribution suitable for use in cement composites.

### 3.2. Gas Emission Study

**Figure 5:**
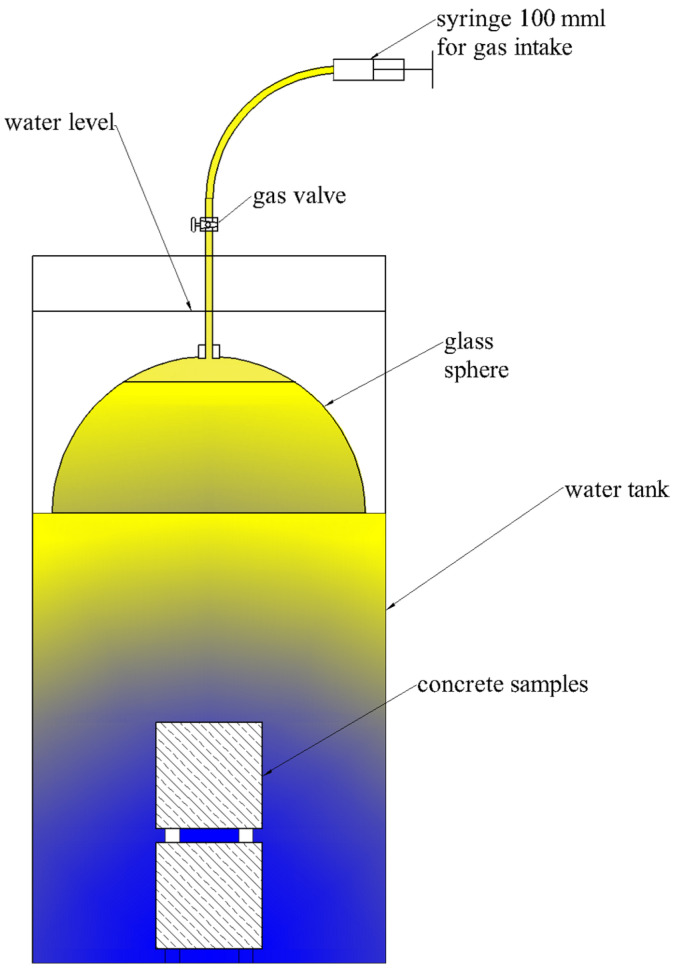
Scheme of the test rig for testing the content of emitted gas.

To measure gas emissions, a test rig and a measurement method were developed. Concrete cubes of known volume (150 mm × 150 mm × 150 mm), 2 in each tank, were submerged in water and covered with glass domes also submerged in water (Figure 5). Concrete cubes were placed in the tank, which came from the formula C (concrete on aggregate composed of slag from waste incineration). The gas emitted from the samples accumulated under the glass dome, and an air/gas cushion was formed. After a certain period of time, depending on the intensity of the gas emission, the accumulated gas was then evacuated with a syringe until the air cushion was completely removed. This made it possible to easily and accurately estimate the volume of gas emitted by the samples.

### 3.3. Chemical Analysis of Concrete Composition

Most of the properties of concrete depend on its structure. The structure of concrete can be analyzed from the atomic level, as well as from the micro- and meso- to the macrometric level, where it is treated as a homogeneous medium. Most often, structure studies are performed for micro- to macrometric levels, where the sizes of distinguishable elements are within the range of 1 μm to 10 mm. Methods of studying the structure of concrete are various and include the analysis of chemical as well as physical processes occurring in its structure. There are several methods for classifying concrete structure testing, such as destructive, non-destructive and semi-destructive methods, optical methods using optical and electron microscopes in reflected and transmitted light, various types of acoustic methods, and finally, X-ray testing [35].

The study used X-ray diffraction, electron spectroscopy, qualitative and quantitative SEM/EDS analysis, and Fourier transform infrared microspectroscopy.

For chemical analysis, cement beams were prepared from milled components whose composition and the tests used are shown in Table 1, items B and C:

- X-ray Diffraction (XRD): The study was conducted using a Bruker (Billerica, MA, USA) AXS D8 Advance X-ray diffractometer with Cu Kα radiation (λ = 1.5406 Å). The scanning was performed in the 2θ range of 5° to 80°, with a step size of 0.02° and a scanning rate of 0.5° per minute to identify the crystalline phases in the cement beams.
- X-ray Photoelectron Spectroscopy (XPS): This analysis was carried out using a multi-chamber ultra-high vacuum (UHV) analytical system from Prevac Ltd. (Sosnowiec, Poland), allowing for detailed surface characterization of the samples.
- Scanning Electron Microscopy (SEM) with Energy Dispersive Spectroscopy (EDS): The SEM/EDS analysis was conducted using a high-resolution environmental scanning electron microscope (Quanta 250 FEG, FEI, Hillsboro, OR, USA) equipped with an EDS analyzer. This technique provided localized elemental mapping and microstructural details of the samples.
- Fourier Transform Infrared (FT-IR) Microspectroscopy: The FT-IR analysis was performed using a Bruker IFS 66/s spectrometer (Billerica, MA, USA), covering the spectral range of 400 to 4000 cm^−1^ with a resolution of 4 cm^−1^. A total of 32 scans were accumulated for each sample to ensure accurate spectral representation.

Optical Emission Spectroscopy (OES): The chemical composition was further analyzed using the MIP OES (Model PLASMAQUANT 100, Carl Zeiss, Oberkochen, Germany). This technique allowed for precise quantification of key elements present in the slag, providing a comprehensive understanding of the material’s composition.

## 4. Results

The results were divided into steps related to the effect of slag on concrete expansion, determining the causes of concrete swelling, and measuring the amount of gasses emitted from the concrete.

### 4.1. Measurements of Gas Emissions from Concrete

The emission of gases, including hydrogen, from concrete made from incinerator slag is a serious technological problem. Concrete cubes 150 mm × 150 mm × 150 mm prepared according to the recipes described in A of Table 1 were tested for gas emissions from the time the samples were unmolded for a period of 167 days. The tests were terminated when the daily gas emissions decreased to approximately 5% of the daily maximum and continued for 5 subsequent days. The laboratory tests conducted showed intense gas emissions during the first 40 days; then, the gas emissions decreased significantly. A side effect was swelling and cracking of the concrete.

**Figure 6:**
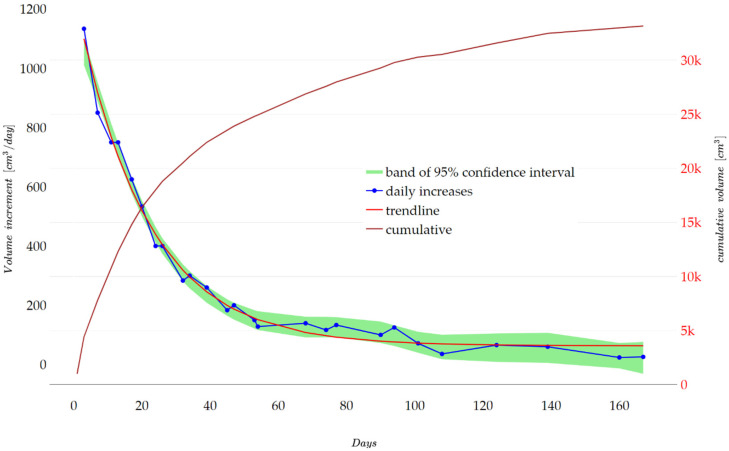
Cumulative gas emissions and daily gas increments from sample A.

Figure 6 shows the cumulative volume of gases emitted by the concrete cubes (2 pieces) in tank one. The figure also shows the changes in the daily increase in the volume of gas released, along with the bands of the 95% confidence interval and the trend line (r^2^ = 0.988).

A total of approximately 33 L of gas was collected during the test.

The collected gas mixture was flammable, indicating a hydrogen content of >4% [36].

### 4.2. Analysis of Chemical Composition of Samples by SEM/EDS and XRD Diffractometer

#### 4.2.1. Sample C

**Figure 7:**
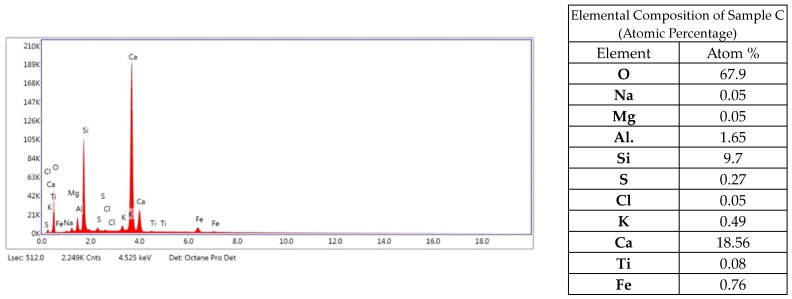
Elemental EDS analysis of sample C.

Analyzing the elemental composition, it can be concluded that the sample mainly consists of oxygen, calcium, aluminum, iron and silicon (Figure 7). Elemental content in the composition of less than 0.5% can be considered insignificant, or their appearance may be due to a measurement error of the instrument.

**Figure 8:**
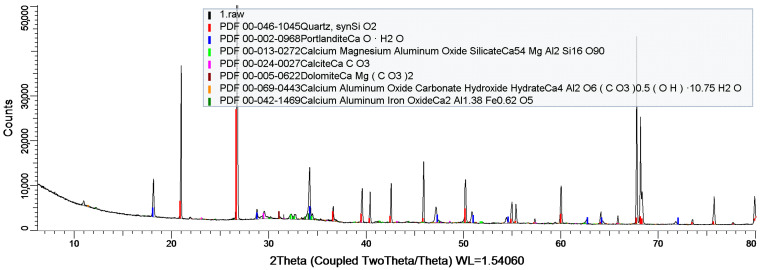
Diffractogram of sample C.

XRD studies showed the formation of crystal structures, i.e., quartz, portlandite, dolomite, calcite, and crystals involving aluminum and iron (Figure 8).

**Figure 9:**
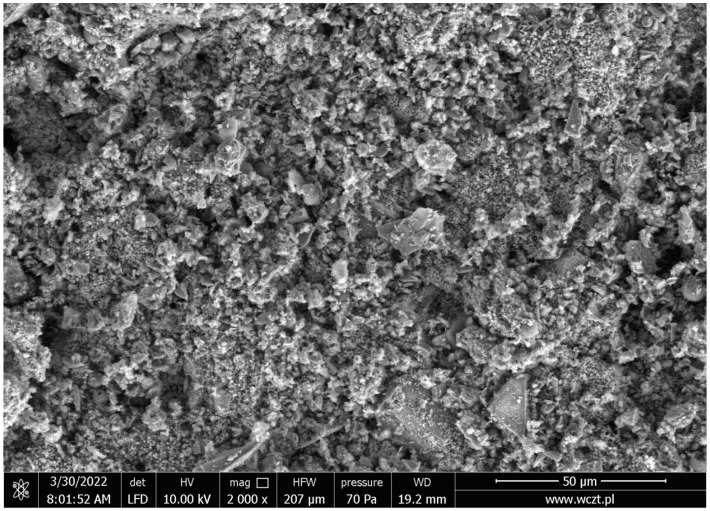
SEM photo of sample C.

Figure 9 shows an image from the SEM analysis of the sample C.

The image shows the formed structure of the C-S-H phase, through which the metal crystals are embedded. The presence of quartz and portlandite can also be seen.

#### 4.2.2. Sample B

**Figure 10:**
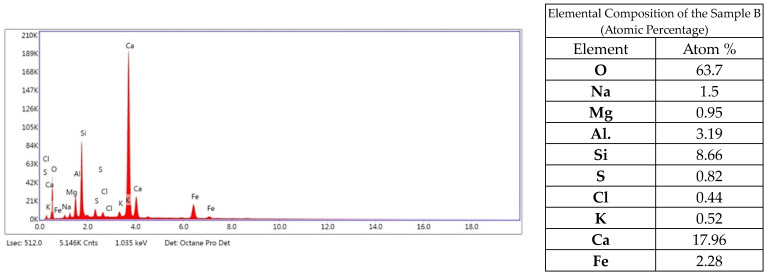
Elemental EDS analysis of sample B.

Analyzing the elemental composition, it can be concluded that the sample mainly consists of oxygen, calcium, aluminum, iron, silicon, magnesium, sodium and sulfur (Figure 10). Elemental content in the composition of less than 0.5% can be considered insignificant, or their appearance may be due to a measurement error of the instrument.

**Figure 11:**
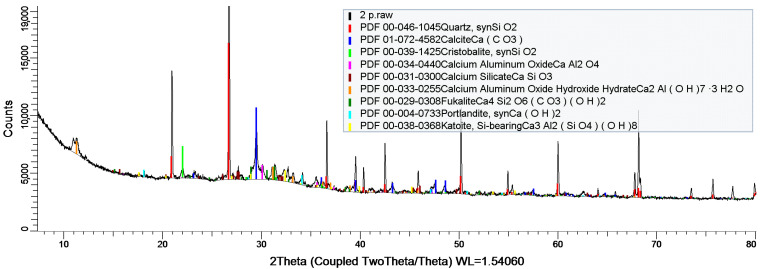
Diffractogram of sample B.

XRD analysis (Figure 11) showed significant amounts of quartz and calcite. Calcite is characterized by a wide variety of crystallographic forms. The presence of aluminum compounds was also confirmed by XRD analysis.

**Figure 12:**
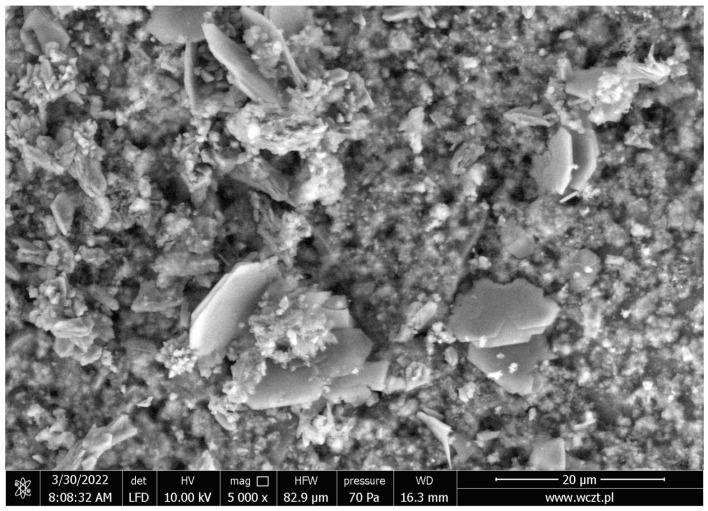
SEM image of sample B.

Aluminum reacts with calcium to form calcium aluminates and hydrated forms. Hydrocalumite, which is typically crystalline, is characterized by hexagonal, plate-like structures, as observed in the SEM image (Figure 12). The hydration mechanism of calcium aluminates is related to the dissolution process in which the anhydrous phase dissolves and the hydrates precipitate from the solvent. Three independent phases can be distinguished: dissolution, nucleation and precipitation. The hydration process is initiated by hydroxylation of the cement surface, followed by dissolution of the cement in water and release of calcium and aluminum ions. A small amount of hydrate gel is formed when the concentration of ions exceeds the solubility level of C_2_AH_8_ and AH_3_ hydrates. Solubility continues with a parallel increase in the concentration of calcium and aluminum ions in the water until a saturation level is reached. Then, crystal spores are formed in large numbers, this phase is called nucleation.

### 4.3. XPS Analysis

**Figure 13:**
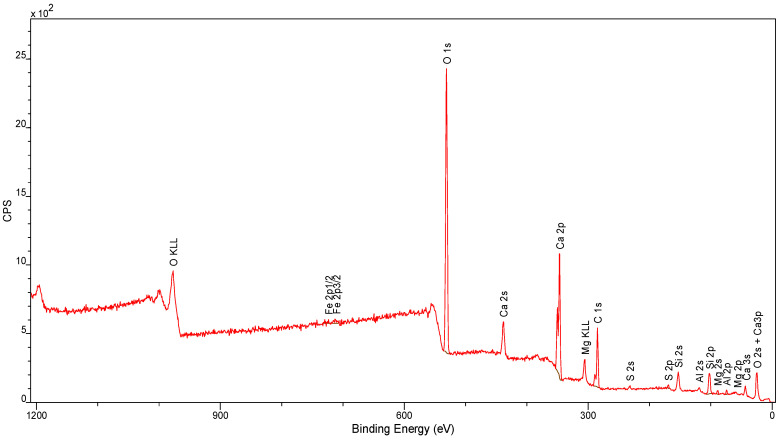
XPS spectra for sample C.

**Figure 14:**
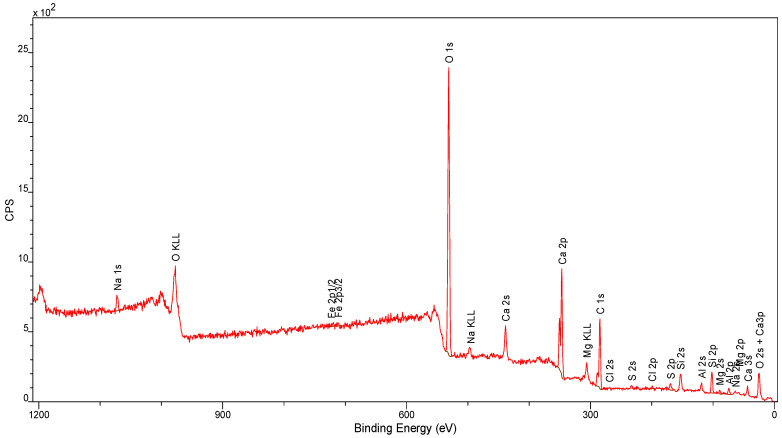
XPS spectra for sample B.

The overall XPS spectra of the various samples analyzed are shown in Figure 13 and Figure 14.

The quantifications of the individual elements analyzed in the samples and their atomic percentages are summarized in Table 2.

For both carboxyl groups and carbonates, the binding energies in the C1s region found in the literature vary within wide limits. Thus, the -COO- peak most likely corresponds to carbonates, and the “carbonates” peak to bicarbonates. This makes chemical sense, since the samples contain alkaline components and have certainly been in contact with atmospheric CO_2_ and water vapor.

The silicon Si2p signal is a doublet: it consists of two peaks 0.6 eV apart, with an area ratio of 1:2. The authors of many works perform the fitting using only one peak. The changes in the observed binding energy here are mainly responsible for the following:

- the degree of oxidation of silicon;
- the degree of hydroxylation;
- structural and chemical factors.

Considering the conditions of cement manufacture, the observed changes in binding energy could be attributed to the latter two factors, with detailed identification from XPS spectra alone being very difficult. It was therefore decided to distinguish only among silica, silica with surface hydroxyl groups, and silicates.

As with carbonates in the C1s region, the reported binding energies in the Ca2p region for calcium carbonate fluctuate within wide limits. One is therefore inclined to attribute a doublet of peaks to carbonates as well as to dicarbonates and sulfates (sulfate sulfur is present in the samples).

An aluminum peak doublet was assigned for oxide and hydroxide forms.

Analysis of XPS spectra of individual samples showed the presence of silica (quartz) and silicic acid salts (silicates), calcium carbonate (calcite), calcium and magnesium carbonate (dolomite), hydroxy aluminates, aluminum oxides and iron oxides, and small amounts of chlorine compounds (except for sample No. C). It is noteworthy that the presence of metals other than iron, aluminum, calcium, sodium and magnesium was not detected, within the detection limit of the equipment used.

### 4.4. FT-IR Analysis

**Figure 15:**
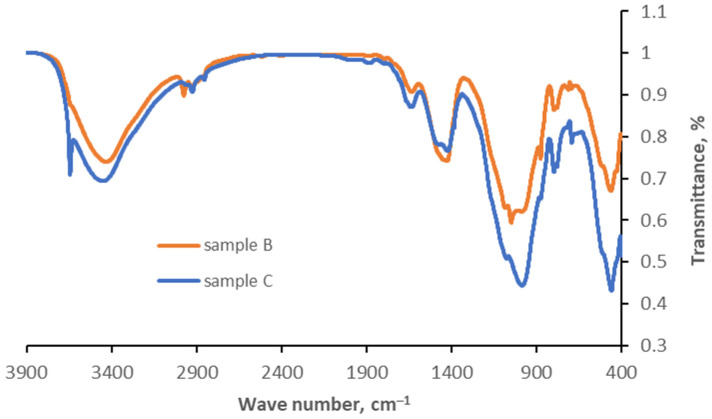
FT-IR spectra for samples B and C.

Analysis of the qualitative composition of the samples based on FT-IR spectra was performed. Figure 15 shows the spectra for each sample tested.

Analysis of the FT-IR spectra showed that the individual absorption bands for all analyzed samples overlap, with peaks occurring at the same wavelength numbers. This shows that all analyzed samples contained a similar composition, the main components of which were carbonates (calcite, dolomite) and quartz and silicates. This confirms the presence of the structures shown by XRD analysis.

The FT-IR spectrum of the analyzed samples showed the presence of absorption bands in the cm^−1^ wave number range:

- 1650–1600 and 3800–3000, originating from hydration water in hydrated compounds;
- 879, 1429–1492, 680—intramolecular vibrations of ${CO}_{3}^{2-}$—anions;
- 1417—symmetric, stretching vibrations of ${CO}_{3}^{2-}$;
- 950–1200—antisymmetric stretching vibrations of Si-O-Si and Si-O-Al;
- 400–550—bending vibrations of O-Si-O;
- 460, 780, 1084—quartz bands.

## 5. Discussion

Energy consumption and carbon dioxide emissions associated with the cement production process, waste disposal problems and the ever-decreasing supply of aggregates in Poland and around the world are forcing the search for and use of alternative concrete compositions.

**Figure 16:**
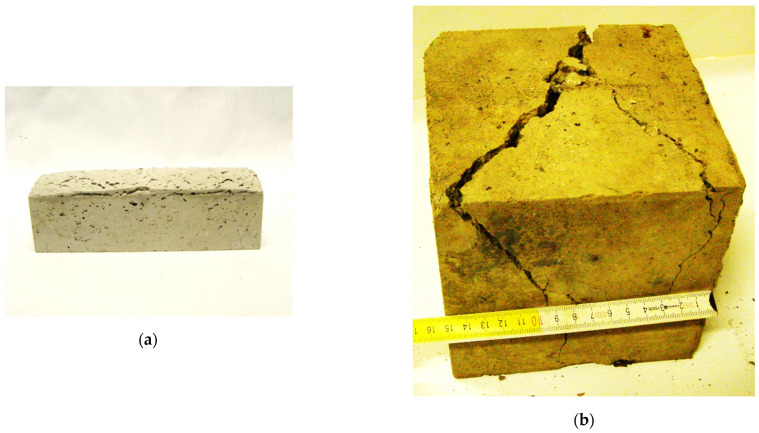
Swollen beam (**a**), and cracks in concrete specimen (**b**) caused by SOK slag supplements.

The slag analyzed in the study contains in its composition both favorable, due to slag stability, tricalcium silicates and harmful aluminum oxides, which in combination with moisture form hydroxides, causing a significant increase in the volume of the cement composite and grain destruction [37]. In all samples containing slag in the composition, the appearance of aluminum was noted, which reacted with calcium and carbon in the presence of water to form hydroxides [38,39]. The result was a significant increase in the volume of the samples, including cement beams by about 7% relative to the volume of the mold (Figure 4), and numerous cracks or bursts of concrete samples (Figure 16).

**Figure 17:**
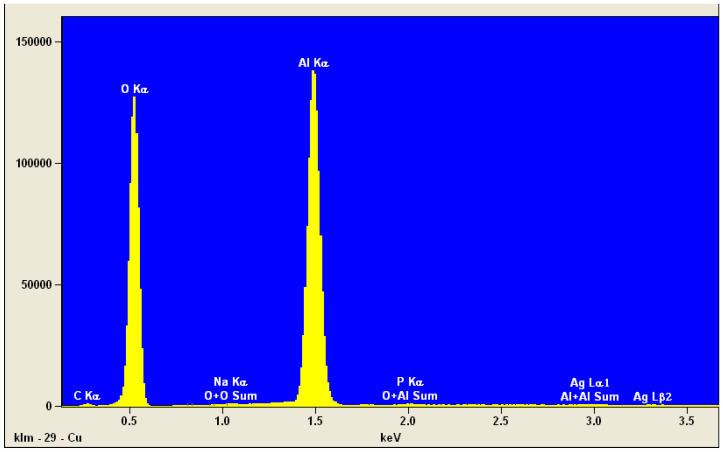
EDS analysis of the swollen portion of the sample.

**Figure 18:**
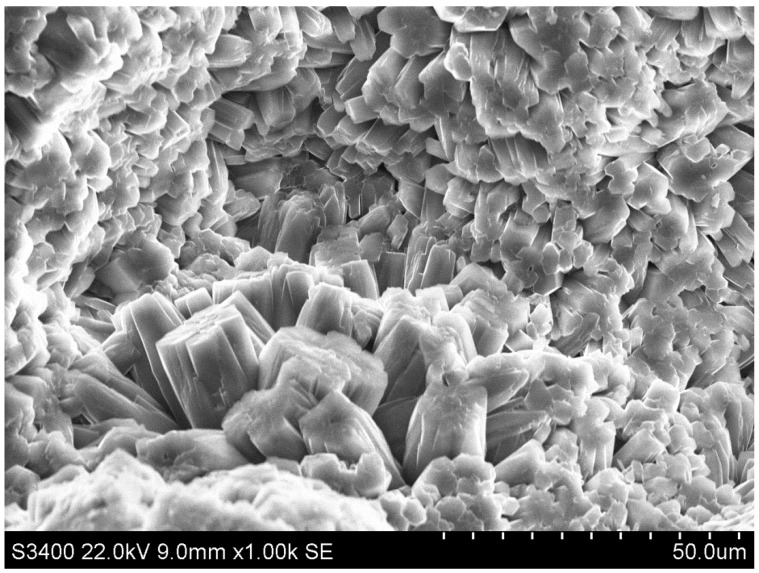
Image of SEM of part of the concrete expansion sample.

SEM/EDS analysis of the swollen sample fragment taken showed the presence of significant amounts of aluminum (Figure 17) and the formation of a tight crystal structure involving it (Figure 18).

In slag, calcium decomposition is also of great importance, i.e., the polymorphic transformation of the calcium orthosilicate, larnite β-C_2_[SiO_4_], which is a metastable variety under normal pressure conditions, to the stable variety γ-C_2_[SiO_4_], which is associated with volume changes [40].

In addition, the appearance of significant amounts of calcite or CaCO_3_ in the samples was observed, which can cause carbonation of concrete.

Another problem observed in the study was the presence of significant amounts of gasses, including hydrogen. The appearance of hydrogen is related to the presence of aluminum in the slag. This is due to the chemical reactions between aluminum and hydroxide ions (OH^−^) in the presence of water (H_2_O), which result in the formation of tetrahydroxyaluminate ions [Al(OH)_4_]^−^ or hexahydroxyaluminate ions [Al(OH)_6_]^3−^ (depending on the pH), accompanied by the release of hydrogen (H_2_).

2Al + 2OH^−^ + 6H_2_O → 2[Al(OH)_4_]^−^ + 3H_2_↑ (tetrahydroxyaluminate)

2Al + 6OH^−^ + 6H_2_O → 2[Al(OH)_6_]^3−^ + 3H_2_↑ (hexahydroxyaluminate)

The volume of the concrete mix increases due to the pressure exerted by the resulting gas [23]. In the study, an increase in volume of about 7% relative to the dimensions of the form was observed.

Mixtures with slag should be stored for at least 5 months, which was confirmed by gas emission tests. After 167 days, a decrease in daily gas emissions to approximately 5% of the recorded maximum daily gas emissions was observed. However, it appears that the stabilization period should be extended to approximately one year under humid conditions [40]. This period is necessary to complete the chemical reactions that result in the release of a significant amount of gas and hydrogen.

## 6. Conclusions

Studies have shown that slag, derived from municipal solid waste incineration, can potentially be used as an aggregate in concrete production. However, due to the presence of unwanted elements, an appropriate processing is therefore necessary to minimize the negative impacts on both the physicochemical, mechanical, and functional properties of cement composites.

The presence of toxic elements in slag poses potential risks to human health and the environment. These elements can impact air and water quality during the production, use, and disposal of building materials [41]. Therefore, it is essential to implement proper processing methods, such as washing, seasoning, or chemical stabilization, before using slag in concrete to reduce the content of harmful substances [42]. While our study focused on the influence of aluminum in slag on concrete structure and hydrogen emission, future research should also consider the long-term environmental and health impacts of using such materials in construction [43].

It has been confirmed that the presence of significant amounts of aluminum in the slag has a substantial impact on the structure of the concrete. This phenomenon occurs due to redox reactions between metallic aluminum and hydroxide ions in the presence of water. These reactions lead to the formation of either tetrahydroxyaluminate ions or hexahydroxyaluminate ions, depending on the pH level, and are accompanied by the release of hydrogen gas. This leads to the swelling and cracking of the concrete. Furthermore, aluminum may also participate in other chemical reactions occurring within the concrete, thereby affecting the strength of the cement composite. Therefore, before utilizing slag in concrete production, it is essential to subject it to appropriate processing, such as washing and heavy metal removal, along with the application of at least a five-month maturation period. Proper processing of the slag reduces the risk of adverse effects such as swelling and cracking of the concrete, thereby enhancing its durability and strength.

The results obtained indicate the necessity for further research and optimization of slag processing methods in order to ensure the safety and durability of concrete. Properly processed slag can contribute to waste reduction while simultaneously promoting sustainable development in the construction industry.

## Figures and Tables

**Table 1:** Test recipes for samples on cement beams and concrete blocks.

	Components	Mass
A		At 32 dm^3^ [kg]
	Slag from SOK 0–2 mm fraction	30.0
	Slag from SOK fraction 2–16 mm	20.31
	Cement CEM I 42.5 N	8.75
	Water	13.46
B	Cement CEM I 42.5 N	450 g
	SOK milled slag	1350 g
	Water	225 cm^3^
C *	Cement CEM I 42.5 N	450 g
	Norm sand	1350 g
	Water	225 cm^3^

* Reference recipe.

**Table 2:** Quantifications of individual elements analyzed in samples.

Sample Identifier	Name	Position eV	Area/(RSF × T × MFP)	%At Conc	% St.Dev.
Sample C	C 1s	284.9	1296.370	24.4	1.26
	O 1s	530.9	2374.050	44.6	1.15
	Mg 2s	88.4	108.297	2.0	0.69
	Al 2p	74.1	119.317	2.2	0.76
	S 2p	169.4	28.282	0.5	0.31
	Ca 2p	346.4	710.252	13.4	0.50
	Fe 2p	713.1	13.716	0.3	0.40
	Si 2p	102.6	671.891	12.6	1.14
Sample B	C 1s	284.3	1576.060	28.9	1.56
	O 1s	531.1	2240.440	41.1	1.45
	Na 1s	1072.6	42.978	0.8	0.42
	Mg 2s	89.3	121.507	2.2	1.20
	Al 2p	74.3	262.221	4.8	1.52
	Si 2p	102.1	512.349	9.4	1.14
	S 2p	169.6	95.835	1.8	0.62
	Cl 2p	199.6	13.326	0.2	0.24
	Ca 2p	346.6	573.933	10.5	0.58
	Fe 2p	719.3	9.783	0.2	0.57

Detailed binding energy values for individual elements, characteristic of the type of bonds formed, are summarized in Table 3, Table 4, Table 5, Table 6 and Table 7.

**Table 3:** Energy values for individual elements.

Name	Position eV	Species	Short Description
O 1s A	529.98	O^−2^	oxide anion (metal oxides)
O 1s B	530.73	O^−2^	calcium oxide
		O^−2^	aluminum oxide
		O=C-O	carboxyl groups
O 1s C	531.85	CaCO_3_	calcium carbonate
		O=C	carbonyl groups
O 1s D	532.92	O-Si	silica
		C-O	C-O (hydroxyls, ethers)

**Table 4:** Energy values for individual elements (carbon).

Name	Position eV	Short Desctiption
C-H	284.70	aliphatic carbon
C=C sp2	284.07	aromatic carbon
C-C sp3	285.29	aliphatic carbon
C-OH	285.93	hydroxyl groups
C-O-C	286.72	epoxy/ether groups
C=O	287.64	carbonyl groups
-COO-	288.71	carboxyl groups
carbonates	289.70	carbonates

**Table 5:** Energy values for individual elements (silicon).

Name	Position eV	Species
Si 2p 3/2 A	101.42	silicates
Si 2p 1/2 A	102.02	
Si 2p 3/2 B	103.08	silica Si-OH
Si 2p 1/2 B	103.68	

**Table 6:** Energy values for individual elements (calcium).

Name	Position eV	Species
Ca 2p 3/2	346.88	CaCO_3_
Ca 2p 1/2	350.44	

**Table 7:** Energy values for individual elements (aluminum).

Name	Position eV
Al 2p Al-O	73.98
Al 2p Al-OH	74.84

## Data Availability

The original contributions presented in this study are included in the article. Further inquiries can be directed to the corresponding author.
